# Identification of a distinct desensitisation gate in the ATP-gated P2X2 receptor

**DOI:** 10.1016/j.bbrc.2019.12.028

**Published:** 2020-02-26

**Authors:** Anastasios Stavrou, Richard J. Evans, Ralf Schmid

**Affiliations:** a Department of Molecular and Cell Biology, University of Leicester, Leicester, LE1 7RH, United Kingdom; b Leicester Institute of Structural and Chemical Biology, University of Leicester, Leicester, LE1 7RH, United Kingdom

**Keywords:** Purinergic signalling, P2X receptors, P2X2, Desensitisation, Electrophysiology, Molecular modelling, P2XR, P2X receptor, MTSET, 2-(Trimethylammonium)ethyl methanethiosulfonate

## Abstract

P2X receptors are trimeric ATP-gated ion channels. In response to ATP binding, conformational changes lead to opening of the channel and ion flow. Current flow can decline during continued ATP binding in a process called desensitisation. The rate and extent of desensitisation is affected by multiple factors, for instance the T18A mutation in P2X2 makes the ion channel fast desensitising. We have used this mutation to investigate whether the gate restricting ion flow is different in the desensitised and the closed state, by combining molecular modelling and cysteine modification using MTSET (2-(Trimethylammonium)ethyl methanethiosulfonate). Homology modelling of the P2X2 receptor and negative space imaging of the channel suggested a movement of the restriction gate with residue T335 being solvent accessible in the desensitised, but not the closed state. This was confirmed experimentally by probing the accessibility of T335C in the P2X2 T18A/T335C (fast desensitisation) and T335C (slow desensitisation) mutants with MTSET which demonstrates that the barrier to ion flow is different in the closed and the desensitised states. To investigate the T18A induced switch in desensitisation we compared molecular dynamics simulations of the wild type and T18A P2X2 receptor which suggest that the differences in time course of desensitisation are due to structural destabilization of a hydrogen bond network of conserved residues in the proximity of T18.

## Highlights

• Ionic gate controls current flow in closed state, gate opens when ATP binds.
• MTSET labelling suggests desensitisation gate in different place than closed state.
• T18A affects desensitisation by disrupting a conserved hydrogen bond network.

## Graphical abstract

**Figure:**
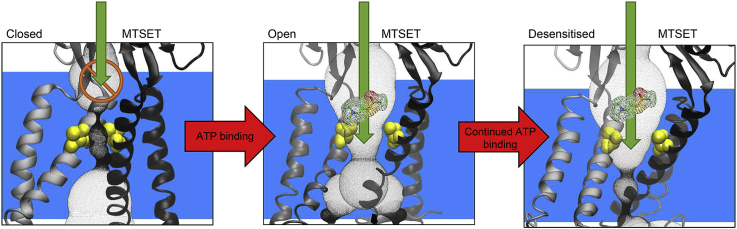

## 1 Introduction

The P2X family of ligand gated ion channels consists of seven paralogs (P2X1-7R) that form trimeric receptors which are activated by ATP. Structurally, P2XRs comprise a large extracellular region which contains the ATP binding sites, a transmembrane region formed by two transmembrane helices per subunit, and a typically small intracellular region formed by the N- and C-terminal regions. While P2XRs share their overall architecture there are differences in ATP sensitivity and time-course of the response to ATP [1]. The P2X1R and P2X3R are activated by low concentrations of ATP (EC_50_ < 1 μM) with currents that decay rapidly during the continued presence of agonist (within 1–2s) in a process called desensitisation. Conversely, the P2X7R has an EC_50_ of >300 μM and currents are sustained and can facilitate on repeated application [2]. The variation in agonist sensitivity is associated with differences in the extracellular ligand binding region of the receptor, for example recent work suggests that a P2X7R specific salt bridge contributes to the high concentrations of ATP required to activate the receptor [3].

The molecular basis of the differences in time-course of P2XR responses was first suggested from work showing that a splice variant in the intracellular C-terminus of the P2X2R could modify the rate of desensitisation and a study using chimeras showing the importance of the intracellular amino terminus of the receptor [4,5]. Subsequent work identified the importance of residues around the conserved YxTxK/R amino terminal sequence motif in determining the time-course of the response [6,7]. P2X2 receptors mutated in this region, such as T18A or T18N, switch the receptor from slowly to quickly desensitising (<1 s) [8].

The crystallisation of the zebrafish P2X4R provided a detailed structural model for ATP binding and opening of the transmembrane channel gate [9,10]. More recently stuctures of the human P2X3R in a range of states became available that provided the first structural information on the desensitised state of the receptor [11]. In the agonist-free, closed state, no structural information could be resolved on the intracellular regions. Two other forms of the hP2X3R resolved an ATP bound state with an open conformation of the ion channel with the intertwining of the N- and C-terminal regions to form an intracellular cap, and a desensitised ATP-bound form where the channel gate had moved and the intracellular cap had disassembled [11]. These studies provided the first structural models of the gating cycle of a P2XR and are consistent with the suggestion that there is a distinct desensitisation gate in the receptor [12].

We therefore devised a strategy to provide experimental evidence for the suggestion that the gate to ionic flow moves in the desensitised state. Pioneering cysteine scanning mutagenesis studies [[13], [14], [15], [16]] provided a good understanding of the location of the channel gate in the P2X2R that is consistent with structural work. One of the key findings in the location of the channel gate was the identification of a cysteine mutant (in hP2X2R; T335C) that blocks ionic flow when chemically modified by methanethiosulfonate compounds in the open, but not in the closed state of the receptor, and hence showed gated access. In this work we tested experimentally whether T335C is accessible in the desensitised state using a mutation in the intracellular amino terminus (T18A) that makes the hP2X2R rapidly desensitising, and we probed computationally how, at a mechanistic level, the T18A mutation might affect the time course of desensitisation.

## 2 Methods

### 2.1 Molecular modelling

Homology models for the human P2X2 receptor were based on the sequence entry GenBank AAD42947.1, which lacks the 12 N-terminal residues of the SwissProt entry P2RX2_HUMAN (Q9UBL9). For modelling the P2X2R open state, the P2X3R open state X-ray structure (PDB identifier: 5SVK), and accordingly, for the P2X2R closed and desensitised state models, PDB entries 5SVJ and 5SVL were used as templates. P2X2R and P2X3R sequences were aligned using MAFFT [17] (pairwise template-target sequence identity ∼ 50%) and manually inspected in Jalview (v2.10.5) [18] to adjust gaps and insertions based on the structure of the respective template [19]. Alignments and templates were used in Modeller v9.19 [20] to calculate 50 models for each state. The resulting models were ranked by their Discrete Optimized Protein Energy (DOPE) score. The best scoring models were used to generate a negative space image of the tunnel between the subunits using HOLE (v2.2.005 on Linux) [21] with a 8 Å pore radius parameter. Calculated channel descriptors were converted to a vmd_plot and visualised together with the receptor in VMD [22]. Pymol [23] was used for additional visualizations.

In total, six molecular dynamics simulations were performed for alternative P2X2R and P2X2R T18A models. Simulations were run in AMBER18 using the ff14SB and lipid17 force fields with the TIP3P water model [24,25] and ATP parameters as in previous work [26]. The set-up involved embedding the P2X2R in a 1-palmitoyl-2-oleoyl-sn-glycero-3-phosphocholine (POPC) lipid bilayer using PACKMOL-Memgen [27] and charge neutralisation with Cl^-^ and Na^+^ at 0.15 M. At this stage, the T18A mutation was introduced into each replicate. The simulation protocol involved 5000 steps steepest descent and 5000 steps of conjugate gradient energy minimization, followed by two heating steps from 0 to 100 K at NVT, and 100K–303K at NPT conditions. A 20 ns holding step to equilibrate the system’s periodic boundary condition dimensions was followed by 200 ns production runs which were analysed in cpptraj [28] for changes in the local environment of key residues in the cytoplasmic cap and visualised in R (Supplemental Figs. S1 and S2).

### 2.2 Molecular biology and electrophysiological recordings

The constructs encoding wild type hP2X2R receptors were available from previous work [6]. Stage V *Xenopus laevis* oocytes were injected with cRNA synthesised with the T7 mMessage mMachine kit (Ambion). P2XR expressing oocytes were stored at 16 °C in ND96 buffer (in mM; NaCl 96, KCl 2, CaCl_2_ 1.8, MgCl_2_ 1, sodium pyruvate 5 and HEPES 5, at pH 7.6) with 50 μgml^−1^ of gentamycin and tetracycline for 3–7 days with the solution changed daily. For electrophysiological recordings the gentamycin and tetracycline were not present and the CaCl_2_ was replaced with 1.8 mM BaCl_2_.

Two electrode voltage clamp recordings were made using a Geneclamp 500B amplifier with a Digidata 1322A A-D convertor and pClamp 8.2 acquisition software (Molecular Devices) at a holding potential of −60 mV. ATP (magnesium salt, Sigma) was applied via a U-tube perfusion system. (2-(Trimethylammonium)ethyl methanethiosulfonate (MTSET) (1 mM) was applied in the bathing solution and/or co-applied with ATP.

### 2.3 Data analysis and statistics

Data are presented throughout as mean ± S.E.M. For peak currents the individual values were normalized to the mean peak current for the WT receptor in the batch of oocytes. Significant differences from WT were calculated by one-way analysis of variance followed by Dunnett’s test using GraphPad Prism 6 (GraphPad software inc).

## 3 Results

**Fig. 1:**
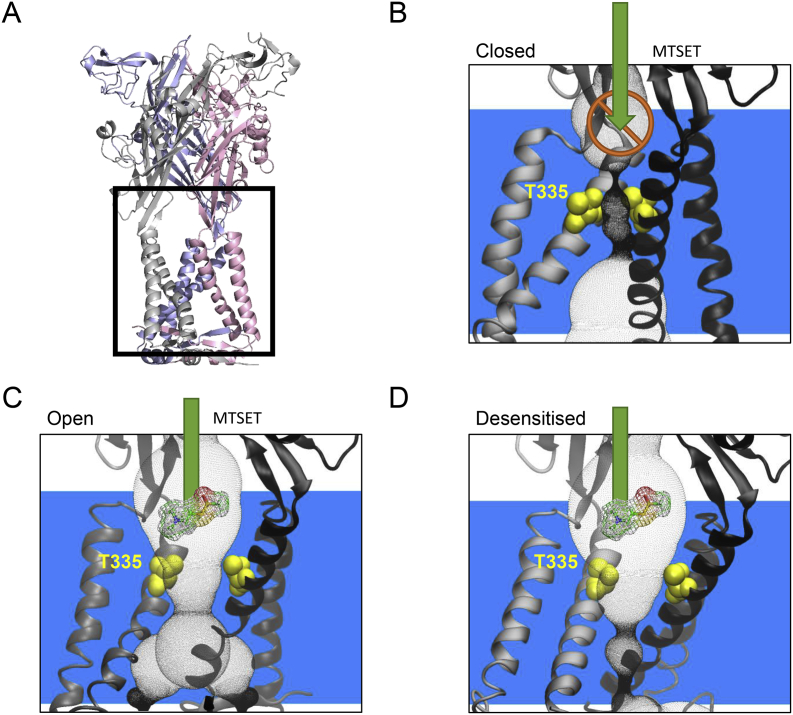
**P2X2R Ionic gate in different conformation states**. (A) hP2X2R homology model in the apo state (closed). The black box in (A) corresponds to the area displayed in other panels indicating the location of the channel gate. In (B), (C), (D) the voids are shown as a grey-dot tunnel. MTSET access through the transmembrane region is blocked by the activation gate near residue T335 (yellow, B). In the open state (C) and the desensitised state (D) residue T335 is accessible.

In previous work on the human P2X1R using voltage clamp fluorometry we showed the kinetics of conformational changes in the extracellular loop associated with desensitisation and suggested that there was a distinct desensitisation gate [12]. Elegant work on P2X2Rs identified pore lining cysteine mutants in TM2 that are accessible only when the channel is ATP bound/open highlighting the location of the activation gate [[13], [14], [15], [16]]. We have extended this work to investigate whether a non-conducting ATP-bound desensitised state involves closure of the activation gate in membrane expressed functional channels. In the P2X2R the T18A mutation [8] switches the receptor from slowly to rapidly desensitising and makes the P2X2R an ideal system to compare the open and desensitised states. As no X-ray structures are available for hP2X2R, we built homology models based on the hP2X3R paralog for the closed, open and desensitised states of the receptor to make predictions that can be tested experimentally (Fig. 1).

In the closed state the transmembrane channel is blocked by the activation gate near residues T335 and T338 (Fig. 1B). Upon ATP binding, the receptor transitions to the open state, (Fig. 1C), as the subunits move outward and the extracellular loop flexes. This flexing causes the TM helices to create a wider pore near the lateral fenestrations [29]. The T335 residues are pulled apart creating a wide opening between the subunits. In the desensitised state (Fig. 1D) T335 residues are even further apart, however at the same time, the V342 residues come closer together to form the desensitisation gate and stop/reduce ion flow.

Cα-Cα distances between T335 residues of different subunits vary between the closed, open and desensitised states ranging from 7.7 Å to 13.2 Å and 14.3 Å, respectively. This indicates changes in the accessibility of T335 to MTS compounds used in this study. Experimentally the accessibility of T335 and hence whether the activation gate is open can be tested by application of MTSET to the T335C mutant. MTSET, like other MTS compounds, can bind covalently to solvent accessible cysteines [30,31]. In case of T335C being accessible to MTSET and oriented towards the pore, covalent binding of MTSET blocks ion flow (see Fig. 1C). We determined how the T18A mutation [8] that makes the hP2X2 receptor rapidly desensitising affects the accessibility of T335C [32].

**Fig. 2:**
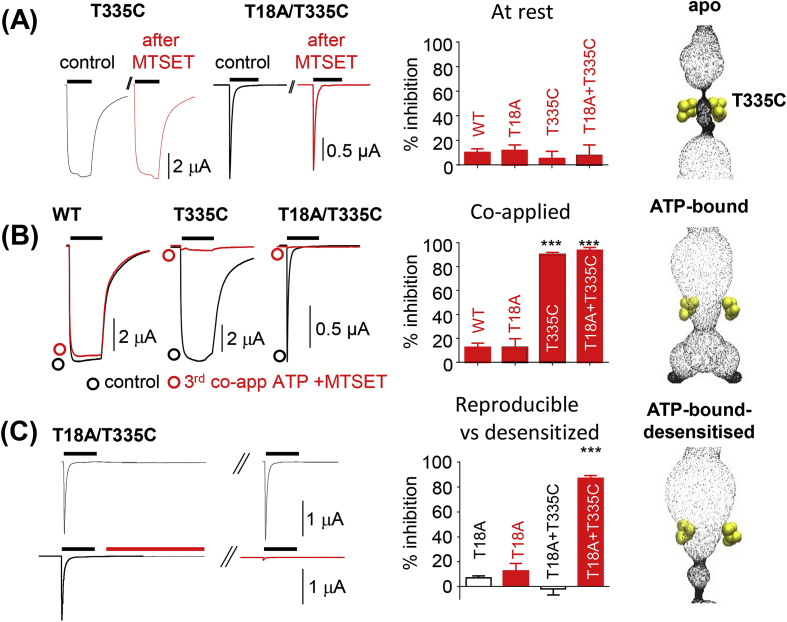
**Effect of MTSET at different stages of ATP-response of P2X2 cysteine mutants.** (A) MTSET (1 mM, 30s) in the absence of ATP had no effect on subsequent ATP currents at WT, T335C or T18A/T335C (fully recovered from desensitisation) showing inaccessibility of T335C in the closed state. Histogram of mean % inhibition of ATP response by MTSET. For all figures black bar indicates ATP application (100 μM, 10 s). (B) MTSET effects when co-applied with ATP showing gated access to residue T335C. There was no effect on the control WT even after the 3rd co-application. Co-application (30s) abolished responses in both the slowly desensitising mutant T335C and the fast desensitising T18A/T335 by more than 90% (C). To determine whether block at T18A/T335C was at the open or desensitised state, MTSET (1 mM, 30s) was applied to the desensitised channel (lower panel). Following washout and the normal period required for recovery from desensitisation (indicated by hashed line, full recovery in control upper panel) subsequent ATP responses were reduced by >90%. Data are shown as mean ± SEM, n = 3–6, ***p < 0.001. The corresponding states of the receptor are indicated on the right-hand side.

Cysteine-reactive MTSET (1 mM), applied for 30 s, in the absence of agonist, had no effect on ATP evoked responses at the hP2X2R T335C mutant and the T18A/T335C double mutant. This demonstrates that T335C is not accessible to MTSET in both systems when they are in the closed state (Fig. 2A). However, when MTSET was co-applied with ATP at the T335C mutant it reduced the response by >95% consistent with previous work [33] indicating that T335C is accessible and can be blocked by MTSET in the open state of the receptor. Under the same conditions the response to ATP for the T18A/T335C fast desensitising mutant was also reduced by >95% (Fig. 2B).

When MTSET was applied for 30 s to the desensitised receptor (T18A/T335C; either in the continued presence, or immediately after removal of ATP, Fig. 2C) the response to a subsequent application of ATP (after a 5 min wash/recovery period) was reduced by >90% demonstrating that T335C is accessible in the desensitised state. In summary our results show that T335C is not accessible to MTSET in the agonist free form, but is accessible in both the ATP-bound open and ATP-bound desensitised states indicating that the activation gate is still open in the desensitised state. This provides the first functional evidence for an additional barrier to ionic permeation in P2XRs, i.e. a desensitisation gate.

**Fig. 3:**
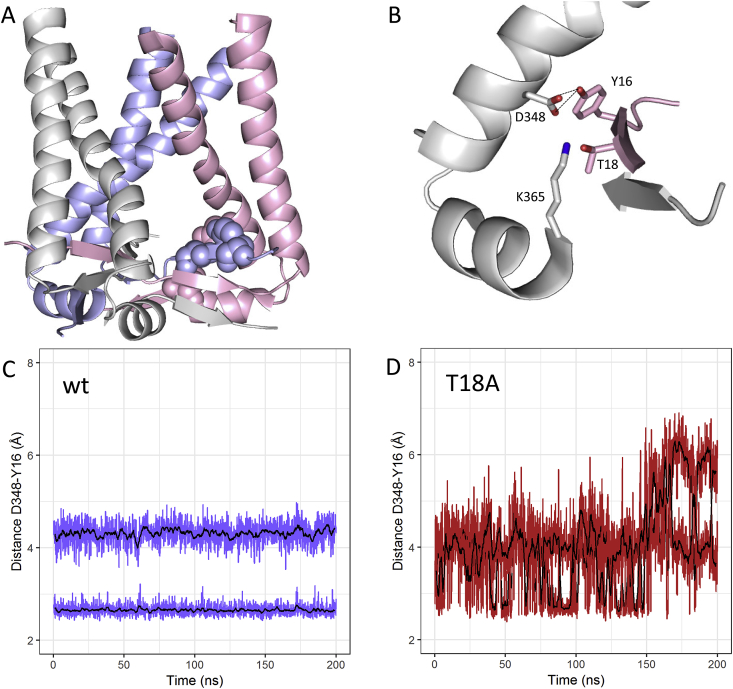
**MD Simulation of P2X2R wildtype and T18A mutant** (A) P2X2R TM and intracellular cap regions in cartoon representation with different subunits in light pink, grey and light blue – colour scheme as in Fig. 1. One instance of residues Y16, T18, D348 and K356 is shown as spheres. (B) Enlarged version of (A) with key residues shown as sticks and dotted lines indicating distance measurements shown in (C) and (D). (C) Representative example for Y16/D348 interaction monitored over a 200 ns molecular dynamics simulations for the P2X2R wildtype. Raw data shown in lightblue and rolling average over 10 data points in black. (D) As in (C) for the P2X2R T18A mutant with raw data shown in firebrick and rolling average over 10 data points in black. (For interpretation of the references to colour in this figure legend, the reader is referred to the Web version of this article.)

We also investigated how the P2X2R T18A mutation may effect the switch from slow to rapid desensitisation. The X-ray structure of the human P2X3R has elucidated a role for T18 (P2X2R numbering) in a framework of conserved residues that link N- and C-terminal regions of the cytoplasmic cap (Fig. 3). For instance, the T18 hydroxy group is in hydrogen bonding distance of the K365 amine (2.3 Å, N–O distance; 5SVK). Similarly, adjacent to T18, the Y16 hydroxy group is in close proximity to the D348 carboxylate (2.2 Å and 4.0 Å, O–O distances; 5SVK). To assess how the T18A mutation affects the stability of the cytoplasmic cap we monitored the distances between the D348 carboxylate and Y16 hydroxy groups in 200 ns molecular dynamics simulations of the human P2X2R with and without T18A mutation (Fig. 3). Due to the trimeric P2XR structrure the tree replicates generated nine time-series for each condition. In the P2X2R wildtype simulations the interaction remained fully stable over the time course (present in >90% of frames) for six of the nine D348/Y16 pairs, in two other pairs it was present in ∼79% and ∼74% of the frames. One time series showed swaps of the interacting carboxylate oxygen combined with intermittent loss of interaction (present in ∼25% of frames). The situation was fundamentally different for the D348 and Y16 interactions in the T18A mutant where none of the time series showed a fully stable D348/Y16 pair, and six of nine pairs were present in less than 40% of frames. In the remaining three time courses the D348/Y16 interaction was present in 67%, 78% and 78% of frames respectively. In summary, this indicates that the T18A mutation affects the dynamics of cytoplasmic cap by destabilising key interactions between conserved residues of adjacent subunits.

## 4 Discussion

***The restriction gate is different in desensitised and closed states.*** Residues lining the P2XR channel gate have been established by cysteine scanning mutagenesis studies well before X-ray structures became available. These were generally consistent with structural biology data (for review see Samways et al.(2014)). In this work we probed experimentally how far the P2X2R ion channel is still open in the desensitised state. This was achieved by using P2X2 wild type and P2X2 T18A receptors to test the accessibility of T335C in the open and desensitised state. MTSET is a water soluble, positively charged MTS-reagent that binds covalently to accessible cysteine residues. In our experimental set-up MTSET is added extracellularly and based on our P2X2R homology models can enter the transmembrane channel via the lower vestibule of the extracellular region of the P2X2R (Fig. 1A). The finding that T335C is not reacting with MTSET in the closed state, but is accessible in open and desensitised states is evidence for the activation gate still being open in the desensitised state, and implies a desensitisation gate as additional barrier to ionic permeation. Our work provides the first direct functional evidence that the channel gate moves in the desensitised state. This is in agreement with cadmium effects on cysteine mutants suggesting that the gate region within TM2 moves during desensitisation [33] and consistent with hP2X3R crystal structures [11].

***Multiple factors contribute to desensitisation.*** Despite the availability of open and desensitised states P2X3R X-ray structures, the molecular mechanism of desensitisation and determinants of different desensitisation patterns in P2X receptor paralogs are not fully understood. It is however clear that different regions of P2X receptors can play a role in desensitisation. For instance, there is evidence from voltage clamp fluorometry that the kinetics of conformational changes in the extracellular region are associated with desensitisation [12]. A role of TM1 and TM2 in desensitisation has been established by TM helix swapping experiments [7], for instance hP2X1R can be made slowly desensitising when either hP2X1R-TM1 or -TM2 of are swappped with equivalent regions [34]. In the intracellular region, changes in intracellular calcium and ATP concentrations [35] and phosphorylation on T372 of the P2X2aR splice variant modulate P2X2R desensitisation [36]. The involvement of extracellular, transmembrane and intracellular regions of P2XRs in the modulation of desensitisation features indicates a multi-faceted process.

***How does T18A affect desensitisation?*** A short stretch of N-terminal residues are conserved throughout the P2XR family (YXTXK/R) and point mutations in this region can have profound effects on time-course of the ATP response. For instance, in P2X2R the T18A mutation transforms the receptor from slowly desensitising to fast desensitising [8]. T18 was originally postulated to be part of a PKC phosphorylation site [8]), though this was later challenged [[37], [38], [39]]. Based on the P2X3R structure in the open state a structural role of the P2X2-T18 region as part of the cytoplasmic cap has emerged, where T18 with other highly conserved residues Y16, D348 and K355 forms an intersubunit hydrogen bond network. The side chains of T18 and Y16 lock the N-terminal beta-strand of the cytoplasmic cap to the C-terminal transmembrane helix via hydrogen bonds to D348, and via K355 to the adjacent short α-helix perpendicular to the membrane (Fig. 3). Our MD simulations suggest that the T18A modification destabilises the tethering between Y16 and D348, and affects structure and dynamics of the cytoplasmic cap. Based on the hP2X3 X-ray structures in different states Mansoor et al. proposed the disassembly of the cytoplasmic cap as key step in desensitisation [11]. In agreement with this proposal the effect of the T18A modification on the dynamics of the conserved H-bond network involving Y16, T18, D348 and K355 we detected in our MD simulations provides a plausible mechanistic explanation how this mutation may accelerate desensitisation.

## Appendix A Supplementary data

Supplemental Table 1 Supplemental Table 1

Suppl Fig. 1.pdf Suppl Fig. 1.pdf

Suppl Fig. 2.pdf Suppl Fig. 2.pdf

## References

[1] Kaczmarek-Hajek K., Lorinczi E., Hausmann R., Nicke A. (2012). Molecular and functional properties of P2X receptors-recent progress and persisting challenges. Purinergic Signal..

[2] Roger S., Gillet L., Baroja-Mazo A., Surprenant A., Pelegrin P. (2010). C-terminal calmodulin-binding motif differentially controls human and rat P2X7 receptor current facilitation. J. Biol. Chem..

[3] Allsopp R.C., Dayl S., Bin Dayel A., Schmid R., Evans R.J. (2018). Mapping the allosteric action of antagonists A740003 and A438079 reveals a role for the left flipper in ligand sensitivity at P2X7 receptors. Mol. Pharmacol..

[4] Brandle U., Spielmanns P., Osteroth R., Sim J., Surprenant A., Buell G., Ruppersberg J.P., Plinkert P.K., Zenner H.-P., Glowatzki E. (1997). Desensitisation of the P2X_2_ receptor controlled by alternative splicing. FEBS (Fed. Eur. Biochem. Soc.) Lett..

[5] Simon J., Kidd E.J., Smith F.M., Chessel I.P., Murrell-Lagnado R., Humphrey P.P.A., Barnard E.A. (1997). Localization and functional expression of splice variants of the P2X2 receptor. Mol. Pharmacol..

[6] Allsopp R.C., Evans R.J. (2011). The intracellular amino terminus plays a dominant role in desensitisation of ATP gated P2X receptor ion channels. J. Biol. Chem..

[7] Hausmann R., Bahrenberg G., Kuhlmann D., Schumacher M., Braam U., Bieler D., Schlusche I., Schmalzing G. (2014). A hydrophobic residue in position 15 of the rP2X3 receptor slows desensitization and reveals properties beneficial for pharmacological analysis and high-throughput screening. Neuropharmacology.

[8] Boue-Grabot E., Archambault V., Seguela P. (2000). A protein kinase C site highly conserved in P2X subunits controls the desensitisation kinetics of P2X2 ATP-gated channels. J. Biol. Chem..

[9] Kawate T., Michel J.C., Birdsong W.T., Gouaux E. (2009). Crystal structure of the ATP-gated P2X(4) ion channel in the closed state. Nature.

[10] Hattori M., Gouaux E. (2012). Molecular mechanism of ATP binding and ion channel activation in P2X receptors. Nature.

[11] Mansoor S.E., Lu W., Oosterheert W., Shekhar M., Tajkhorshid E., Gouaux E. (2016). X-ray structures define human P2X3 receptor gating cycle and antagonist action. Nature.

[12] Fryatt A.G., Evans R.J. (2014). Kinetics of conformational changes revealed by voltage-clamp fluorometry give insight to desensitization at ATP-gated human P2X1 receptors. Mol. Pharmacol..

[13] Rassendren F., Buell G., Newbolt A., North R.A., Surprenant A. (1997). Identification of amino acid residues contributing to the pore of a P2X receptor. EMBO J..

[14] Egan T.M., Haines W.R., Voigt M.M. (1998). A domain contributing to the ion channel of ATP-gated P2X2 receptors identified by the substituted cycteine accessibitlity method. J. Neurosci..

[15] Li M., Chang T.H., Silberberg S.D., Swartz K.J. (2008). Gating the pore of P2X receptor channels. Nat. Neurosci..

[16] Kracun S., Chaptal V., Abramson J., Khakh B.S. (2010). Gated access to the pore of a P2X receptor: structural implications for closed-open transitions. J. Biol. Chem..

[17] Katoh K., Standley D.M. (2013). MAFFT multiple sequence alignment software version 7: improvements in performance and usability. Mol. Biol. Evol..

[18] Waterhouse A.M., Procter J.B., Martin D.M.A., Clamp M., Barton G.J. (2009). Jalview Version 2--a multiple sequence alignment editor and analysis workbench. Bioinformatics.

[19] Stavrou A., Dayl S., Schmid R. (2020). Homology modeling of P2X receptors. Methods Mol. Biol..

[20] Webb B., Sali A. (2016). Comparative protein structure modeling using MODELLER. Curr. Protein Pept. Sci..

[21] Smart O.S., Neduvelil J.G., Wang X., Wallace B.A., Sansom M.S.P. (1996). HOLE: a program for the analysis of the pore dimensions of ion channel structural models. J. Mol. Graph..

[22] Humphrey W., Dalke A., Schulten K. (1996). VMD: visual molecular dynamics. J. Mol. Graph..

[23] Schrödinger L. (2017). The PyMOL Molecular Graphics System.

[24] Maier J.A., Martinez C., Kasavajhala K., Wickstrom L., Hauser K.E., Simmerling C. (2015). ff14SB: improving the accuracy of protein side chain and backbone parameters from ff99SB. J. Chem. Theory Comput..

[25] Case D.A. (2017). AMBER 2017.

[26] Fryatt A.G., Dayl S., Cullis P.M., Schmid R., Evans R.J. (2016). Mechanistic insights from resolving ligand-dependent kinetics of conformational changes at ATP-gated P2X1R ion channels. Sci Rep-Uk.

[27] Schott-Verdugo S., Gohlke H. (2019). PACKMOL-memgen: a simple-to-use, generalized workflow for membrane-protein–lipid-bilayer system building. J. Chem. Inf. Model..

[28] Roe D.R., Cheatham T.E. (2013). PTRAJ and CPPTRAJ: software for processing and analysis of molecular dynamics trajectory data. J. Chem. Theory Comput..

[29] Browne L.E., Jiang L.H., North R.A. (2010). New structure enlivens interest in P2X receptors. Trends Pharmacol. Sci..

[30] Akabas M.H., Stauffer D.A., Xu M., Karlin A. (1992). Acetylcholine receptor channel structure probed in cysteine-substitution mutants. Science.

[31] Roberts J.A., Evans R.J. (2007). Cysteine substitution mutants give structural insight and identify ATP binding and activation sites at P2X receptors. J. Neurosci..

[32] Samways D.S., Li Z., Egan T.M. (2014). Principles and properties of ion flow in P2X receptors. Front. Cell. Neurosci..

[33] Li M., Kawate T., Silberberg S.D., Swartz K.J. (2010). Pore-opening mechanism in trimeric P2X receptor channels. Nat. Commun..

[34] Allsopp R.C., Farmer L.K., Fryatt A.G., Evans R.J. (2013). P2X receptor chimeras highlight roles of the amino terminus to partial agonist efficacy, the carboxyl terminus to recovery from desensitization, and independent regulation of channel transitions. J. Biol. Chem..

[35] Rokic B.M., Castro P., Leiva-Salcedo E., Tomic M., Stojilkovic S.S., Coddou C. (2018). Opposing roles of calcium and intracellular ATP on gating of the purinergic P2X2 receptor channel. Int. J. Mol. Sci..

[36] Coddou C., Sandoval R., Castro P., Lazcano P., Hevia M.J., Rokic M., Hall B., Terse A., Gonzalez-Billault C., Kulkarni A.B., Stojilkovic S.S., Utreras E. (2017). Cyclin-dependent kinase 5 modulates the P2X2a receptor channel gating through phosphorylation of C-terminal threonine 372. Pain.

[37] Brown D.A., Yule D.I. (2007). Protein kinase C regulation of P2X3 receptors is unlikely to involve direct receptor phosphorylation. Biochim. Biophys. Acta.

[38] Franklin C., Braam U., Eisele T., Schmalzing G., Hausmann R. (2007). Lack of evidence for direct phosphorylation of recombinantly expressed P2X(2) and P2X (3) receptors by protein kinase C. Purinergic Signal..

[39] Roberts J.A., Bottrill A.R., Mistry S., Evans R.J. (2012). Mass spectrometry analysis of human P2X1 receptors; insight into phosphorylation, modelling and conformational changes. J. Neurochem..

