# Supplementary material for: Identification of a distinct desensitisation gate in the ATP-gated P2X2 receptor
Source: Biochem Biophys Res Commun. 2020 Feb 26;523(1):190–5. doi: 10.1016/j.bbrc.2019.12.028 (PMC7008354; doi:10.1016/j.bbrc.2019.12.028)
Supplement: Supplemental Table 1 [file mmc1.docx]

| Wildtype and T18A mutant: replicates 1-3, instances A, B and C. | Frames (out of 2000) with Y16(O)-D348(O) distance < 3Å | % |
| --- | --- | --- |
| Wt-1A | 1984 | 99 |
| Wt-1B | 1984 | 99 |
| Wt-1C | 490 | 25 |
| Wt-2A | 1925 | 96 |
| Wt-2B | 1856 | 93 |
| Wt-2C | 1928 | 96 |
| Wt-3A | 1581 | 79 |
| Wt-3B | 1470 | 74 |
| Wt-3C | 1860 | 93 |
|  |  |  |
| T18A-1A | 4 | 0 |
| T18A-1B | 743 | 37 |
| T18A-1C | 1343 | 67 |
| T18A-2A | 556 | 28 |
| T18A-2B | 1760 | 88 |
| T18A-2C | 128 | 6 |
| T18A-3A | 1757 | 88 |
| T18A-3B | 710 | 36 |
| T18A-3C | 191 | 10 |

Supplemental Table 1. Distance measurements between Y16 and D348 over six 200ns MD simulations for P2X2R wildtype and P2X2A T18A.
