# Supplementary material for: Identification of a distinct desensitisation gate in the ATP-gated P2X2 receptor
Source: Biochem Biophys Res Commun. 2020 Feb 26;523(1):190–5. doi: 10.1016/j.bbrc.2019.12.028 (PMC7008354; doi:10.1016/j.bbrc.2019.12.028)
Supplement: Suppl Fig. 1.pdf [file mmc2.pdf]

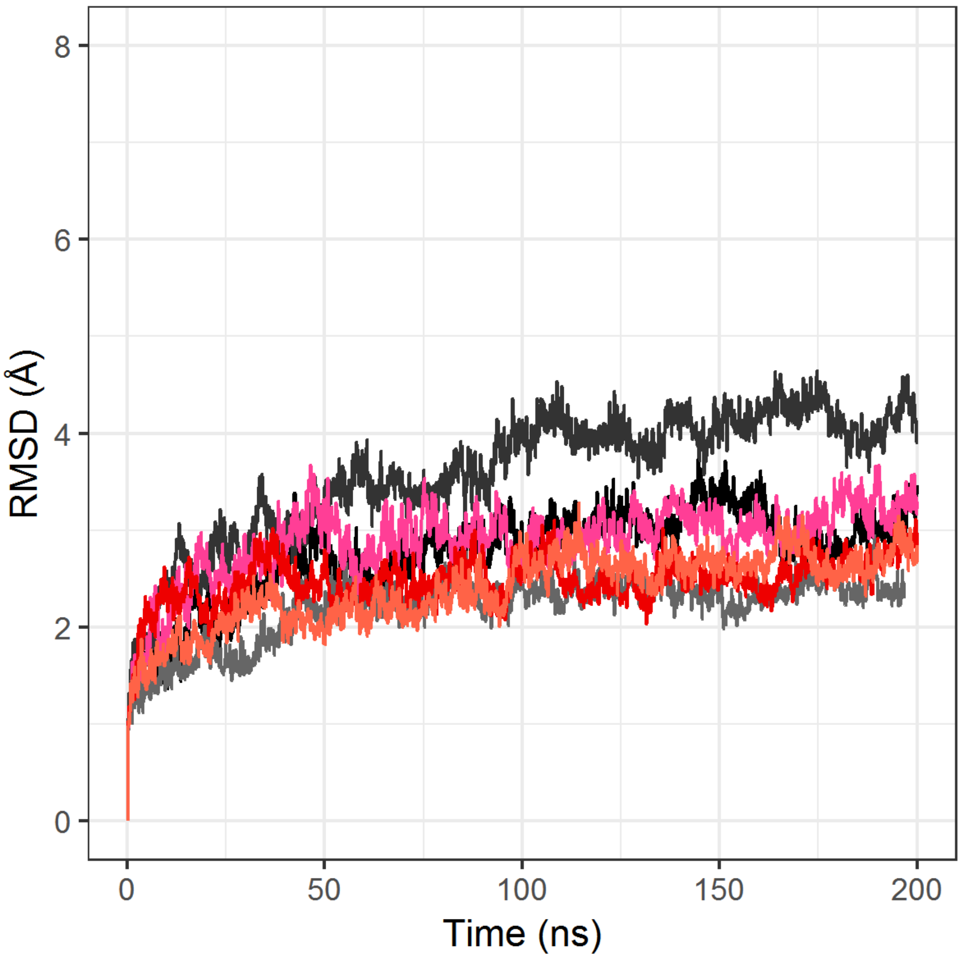

8
6
4
2
0
RMSD (Å)
0
50
100
150
200
Time (ns)

Supplemental Figure 1. RMSD plots for molecular dynamics simulations of the P2X2R wildtype and T18A mutant. The P2X2R wild type simulations are shown in black and grey, P2X2R T18A simulations in different shades of red.
