# Supplementary material for: Identification of a distinct desensitisation gate in the ATP-gated P2X2 receptor
Source: Biochem Biophys Res Commun. 2020 Feb 26;523(1):190–5. doi: 10.1016/j.bbrc.2019.12.028 (PMC7008354; doi:10.1016/j.bbrc.2019.12.028)
Supplement: Suppl Fig. 2.pdf [file mmc3.pdf]

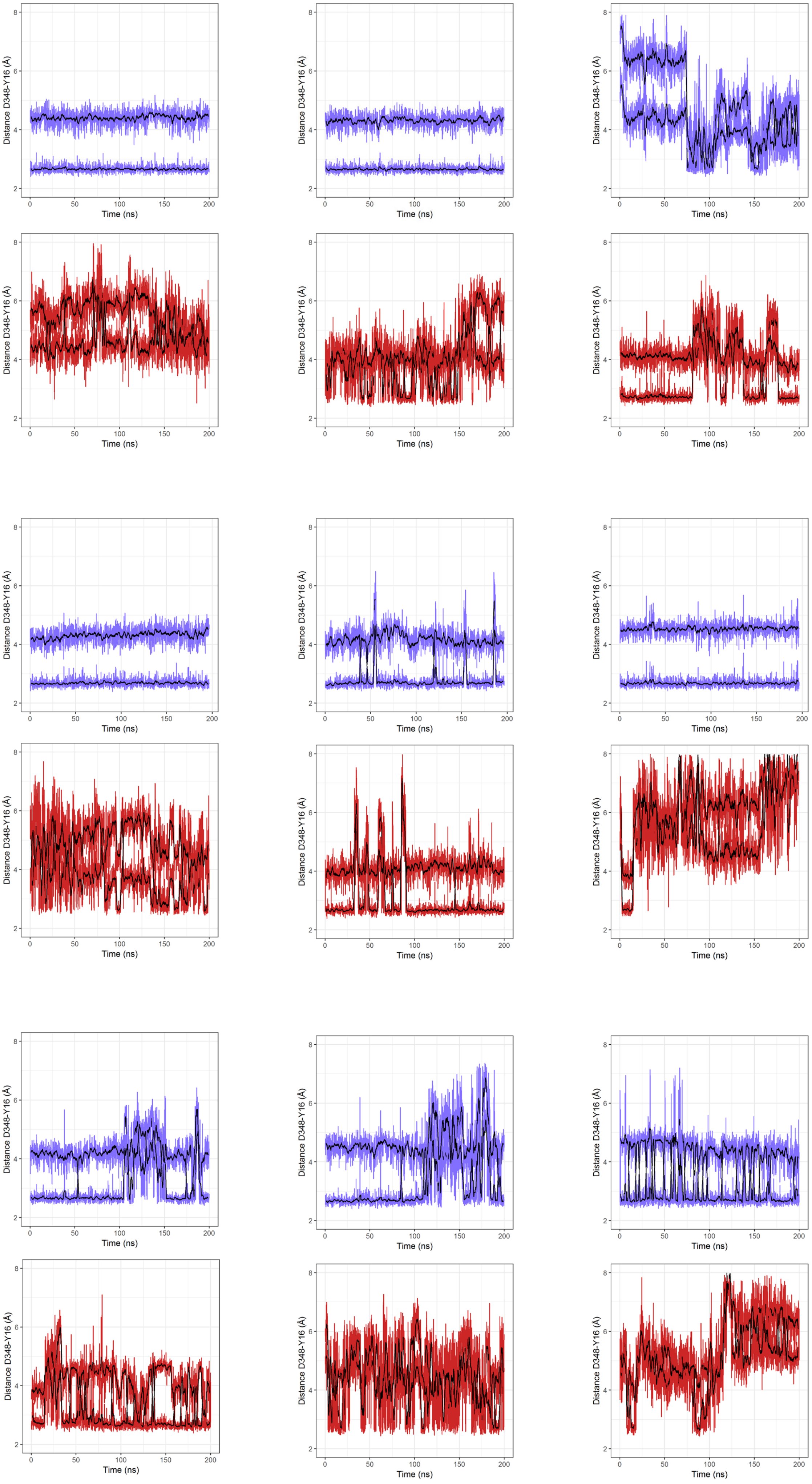
Distance D348-Y16 (Å)
Time (ns)

Supplemental Figure 2. **MD Simulation of P2X2R wildtype and T18A mutant.** Y16/D348 interactions monitored over 200 ns molecular dynamics simulations for the P2X2R wildtype and the P2X2R T18A mutant. Raw data are shown in light blue (wild type) and firebrick red (T18A mutant). Rolling averages over 10 data points are shown in black.
